# Prevalence and determinants of adolescent pregnancy in Africa: a systematic review and Meta-analysis

**DOI:** 10.1186/s12978-018-0640-2

**Published:** 2018-11-29

**Authors:** Getachew Mullu Kassa, A. O. Arowojolu, A. A. Odukogbe, Alemayehu Worku Yalew

**Affiliations:** 1 Pan African University Life and Earth Sciences Institutes, Department of Obstetrics and Gynaecology, College of Medicine, University of Ibadan, Ibadan, Nigeria; 2 Department of Obstetrics and Gynaecology, College of Medicine, University College Hospital, University of Ibadan, Ibadan, Nigeria; 3 School of Public Health, College of Health Sciences, Addis Ababa University, Addis Ababa, Ethiopia; 4 College of Health Sciences, Debre Markos University, P.O.BOX: 269, Debre Markos, Ethiopia

**Keywords:** Adolescent pregnancy, Sociodemographic factors, Systematic review, Meta-analysis, Sub-Saharan Africa, Africa

## Abstract

**Background:**

Adolescence is the period between 10 and 19 years with peculiar physical, social, psychological and reproductive health characteristics. Rates of adolescent pregnancy are increasing in developing countries, with higher occurrences of adverse maternal and perinatal outcomes. The few studies conducted on adolescent pregnancy in Africa present inconsistent and inconclusive findings on the distribution of the problems. Also, there was no meta-analysis study conducted in this area in Africa. Therefore, this systematic review and meta-analysis were conducted to estimate the prevalence and sociodemographic determinant factors of adolescent pregnancy using the available published and unpublished studies carried out in African countries. Also, subgroup analysis was conducted by different demographic, geopolitical and administrative regions.

**Methods:**

This study used a systematic review and meta-analysis of published and unpublished studies in Africa. Preferred Reporting Items for Systematic Reviews and Meta-Analyses (PRISMA) guideline was strictly followed. All studies in MEDLINE, PubMed, Cochrane Library, EMBASE, Google Scholar, CINAHL, and African Journals Online databases were searched using relevant search terms. Data were extracted using the Joanna Briggs Institute tool for prevalence studies. STATA 14 software was used to perform the meta-analysis. The heterogeneity and publication bias was assessed using the *I*^*2*^ statistics and Egger’s test, respectively. Forest plots were used to present the pooled prevalence and odds ratio (OR) with 95% confidence interval (CI) of meta-analysis using the random effect model.

**Result:**

This review included 52 studies, 254,350 study participants. A total of 24 countries from East, West, Central, North and Southern African sub-regions were included. The overall pooled prevalence of adolescent pregnancy in Africa was 18.8% (95%CI: 16.7, 20.9) and 19.3% (95%CI, 16.9, 21.6) in the Sub-Saharan African region. The prevalence was highest in East Africa (21.5%) and lowest in Northern Africa (9.2%). Factors associated with adolescent pregnancy include rural residence (OR: 2.04), ever married (OR: 20.67), not attending school (OR: 2.49), no maternal education (OR: 1.88), no father’s education (OR: 1.65), and lack of parent to adolescent communication on sexual and reproductive health (SRH) issues (OR: 2.88).

**Conclusions:**

Overall, nearly one-fifth of adolescents become pregnant in Africa. Several sociodemographic factors like residence, marital status, educational status of adolescents, their mother’s and father’s, and parent to adolescent SRH communication were associated with adolescent pregnancy. Interventions that target these factors are important in reducing adolescent pregnancy.

## Plain English summary

Adolescent pregnancy is defined as the occurrence of pregnancy in girls aged 10 to 19. Adolescent pregnancy has become a major public health problem, particularly in Africa. Consequently, the region is known for the high rate of maternal and child morbidity and mortality. Since recent times, several governmental and non-governmental organization in some African countries focused on reducing the adolescent pregnancy rate, although a very slow progress was made. Several published and unpublished studies conducted on the prevalence of adolescent pregnancy in Africa are available. However, these studies present inconsistent and inconclusive findings and little is known about the overall epidemiology of adolescent pregnancy in the continent. This study, therefore, was conducted to estimate the prevalence and sociodemographic factors associated with adolescent pregnancy in Africa using published and unpublished studies. This study included a total of 52 studies from 24 African countries. Accordingly, almost one-fifth (18.8%) adolescent get pregnant in Africa. A higher prevalence was observed in East African sub-region (21.5%). Adolescents from rural areas, ever married, whose mother or father were not educated, and had no parent to child communication on SRH issues were more likely to start childbearing at a younger age. Therefore, African countries and other non-governmental organizations need to address these factors and the multifaceted sexual and reproductive health needs of adolescents. Programs aimed at improving the contraceptive use, prevention of unintended pregnancy, prevention of early marriage and risk behavior reduction can reduce the high rate of adolescent pregnancy in Africa.

## Background

Globally, around 1 in 6 people are adolescents aged 10 to 19 years old [1]. Adolescent pregnancy is defined as the occurrence of pregnancy in girls aged 10–19 [2]. Almost one-tenth of all births are to women below 20 years old, and more than 90% of such births occur in developing countries [1, 3]. The declining age at menarche and better nutrition and healthier lifestyles of younger generations are the main factors for high rate of adolescent pregnancy globally [4]. World Health Organization (WHO) 2014 report showed that the global adolescent birth rate was 49 per 1000 girls aged 15 to 19 years old [5].

Adolescent pregnancy is a major public health problem, particularly in Africa [6]. It is associated with high maternal and child morbidity and mortality and affects the socio-economic development of a country [1, 5, 7]. It is linked to an increased risk of adverse pregnancy and childbirth outcomes compared to older women [6]. More than 70,000 adolescent girls die every year because of these complications mainly in developing countries [3]. Most maternal and child morbidity and mortality are related to hypertensive disorders of pregnancy, infections, low birth weight, and preterm delivery [2].

Pregnancy among adolescent women has implications on the educational opportunity, population growth and ill-health of women. For this reason, prevention of child marriage and reduction of adolescent pregnancy has long been the focus of attention by several governmental and non-governmental organizations [8]. Moreover, the reduction in the adolescent pregnancy birth rate since 1990 has resulted in the decline of maternal mortality rate among teenagers especially in developed nations [1]. Several studies have shown that the high level of maternal and perinatal morbidity and mortality can be reduced by lowering the high rate of adolescent pregnancy in developing countries [6, 9, 10]. Consequently, reducing the high rate of adolescent pregnancy and maternal mortality is considered as the key Sustainable Development Goals (SDG),target 3.1 and 3.7 [11].

Even though the identification of the distribution of adolescent pregnancy is important in designing proper interventions to reduce the problem, the small sample sizes and a limited number of available studies were the challenges in identifying the magnitude of the problem in Africa. There is also the absence of the distribution of the problem in different geopolitical and administrative areas. Additionally, the available studies which assessed the factors associated with adolescent pregnancy in Africa showed inconsistent findings [12–25]. Therefore, this review used the evidence of these studies and summarized the pooled estimates using a meta-analysis. There was one previous systematic review [26] conducted to assess the determinants of adolescent pregnancy in Sub-Saharan African countries. However, it included the sociocultural, economic and environmental factors which affect adolescent pregnancy, and didn’t use meta-analysis methods to pool the prevalence and determinants of adolescent pregnancy. The current study, therefore, used both systematic and meta-analysis methods to estimate the pooled prevalence and sociodemographic determinants of adolescent pregnancy in Africa. This study further examined the prevalence of adolescent pregnancy by different study characteristics like sub-regions of Africa, study design and type, publication year, and study quality score. The findings of this study will help to design strategies aimed at reducing adolescent pregnancy and monitor the progress of programs aimed at achieving the adolescent pregnancy rate and maternal mortality reduction targets of SDG.

## Methods

### Study design and search strategy

A systematic review and meta-analysis of published and unpublished studies were conducted to assess the pooled prevalence and associated factors of adolescent pregnancy in Africa. Preferred Reporting Items for Systematic Reviews and Meta-Analyses (PRISMA) guidelines [27] were strictly followed in doing this review. The databases used to search for studies were: MEDLINE, PUBMED, Cochrane Library, EMBASE, Google Scholar, CINAHL, and African Journals Online (AJOL). All search terms for “*Adolescent pregnancy OR teen pregnancy OR teenage pregnancy OR young maternal age AND Africa*” were used separately and in combination using the Boolean operators like “OR” or “AND”. Also, terms like “*determinant factors OR determinant variables OR associated factors”* were used in combination with the above search terms. The search was also made by combining the above search terms with the name of all countries included in Africa.

### Study selection and eligibility criteria

All available studies conducted between 1990 to September 2018 were included in this review. All prospective and retrospective cohort studies, cross-sectional studies, case control and Demographic and Health Survey (DHS) reports of African countries were included in this review. For the latter, only the recent DHS final reports published in the English language were extracted from the official website of the DHS program [28]. The references of the selected articles were also screened to retrieve any additional articles which could be incorporated in this review. However, studies conducted among the non-adolescent population or on male adolescents (teenage fatherhood), or those not reporting the outcome of interest, and review articles were excluded.

### Definition of adolescent pregnancy

The DHS reports measured teenage pregnancy as “*Percentage of women aged 15-19 who have given birth or are pregnant with their first child*” [29–46]. Prevalence of adolescent pregnancy can also be measured as the percentage of pregnant adolescent women from all women who attended health institutions for delivery services [47–57] or antenatal care services [58, 59] during a specific period of time. Therefore, this review included all studies which used either of the above- definitions.

Several sociodemographic related factors which affect adolescent pregnancy were included. These factors include residence (rural vs urban), marital status (ever married vs never married), educational status of an adolescent girl (not attended vs attended), educational status of mother (not attended vs attended) and educational status of the father (not attended and attended). Adolescent girls who were currently married or divorced or previously married or living together were categorized as ever married. Also, for the educational status of adolescents and their family, primary, secondary or tertiary educational levels were grouped as attended and those who have never been admitted to school were grouped as not attended. In addition, parent to adolescent communication on sexual and reproductive health (SRH) issues was also included.

### Quality assessment and data extraction

Articles were screened using their titles, abstracts, and full paper reviews prior to including in the meta-analysis. The Joanna Briggs Institute (JBI) critical appraisal checklist [60] was used to assess the quality of included studies. The tool contains information on sample representativeness of the target population, participant recruitment, adequacy of the sample size, detailed description of the study subjects and study setting, sufficient coverage of the data analysis, objective criteria in the measurement of the outcome variable and identification of subpopulation, reliability, appropriate statistical analysis, and identification of confounding variables. The quality scores of included studies were assessed and presented using the mean scores to designate as high or low-quality.

The JBI tool for prevalence studies [61] was used as a guideline for data extraction from the finally selected articles. The data extraction tool contains information on the author and year of the study, title, year study was conducted and year of publication, study area and country, sub-region, study design and type, study population, age range of adolescent participants, sample size, response rate, the outcome measured, and prevalence rate of adolescent pregnancy. Information regarding the publication status was also collected. Additionally, for the factors, a separate data extraction tool was prepared. The tool contains information on author’s name, year of publication, number of pregnant adolescents and total adolescents by residence, marital status, adolescent’s and their family educational status, and parent to adolescent communication on SRH issues was collected.

### Heterogeneity and publication Bias

The heterogeneity test of included studies was assessed by using the *I*^*2*^ statistics. The *p*-value for *I*^*2*^ statistics less than 0.05 was used to determine the presence of heterogeneity. Low, moderate and high heterogeneity was assigned to *I*^*2*^ test statistics results of 25, 50, and 75% respectively [62]. The publication bias was assessed using the Egger regression asymmetry test [63, 64]. For meta-analysis results which showed the presence of publication bias (Egger test = *p* < 0.05), the Duval and Tweedie nonparametric trim and fill analysis using the random effect analysis was conducted to account for publication bias [65].

### Statistical methods and analysis

Data were entered into Microsoft Excel and the meta-analysis was conducted using STATA 14 software. Forest plots were used to show the magnitude of adolescent pregnancy in Africa. Due to its help in minimizing the heterogeneity of included studies, the random effect model of analysis was used as a method of meta-analysis [62].

Subgroup analyses were also conducted by different study characteristics such as sub-regions of Africa (East, South, West, Central and Northern Africa), study design (cross-sectional or retrospective study), study type (community based or institution based), type of the document (DHS report or research article), publication status (published or unpublished), publication year (before 2015 or after 2015) and study quality score (low or high score). Moreover, the meta - analysis regression was conducted to identify the sources of heterogeneity among studies [66]. It was conducted using the following study-level covariates: sample size, publication year, study quality score, sub-region, and publication status of included studies. The different factors associated with adolescent pregnancy were presented using odds ratios (ORs) with 95% confidence interval (CI).

## Results

### Study selection

**Fig. 1:**
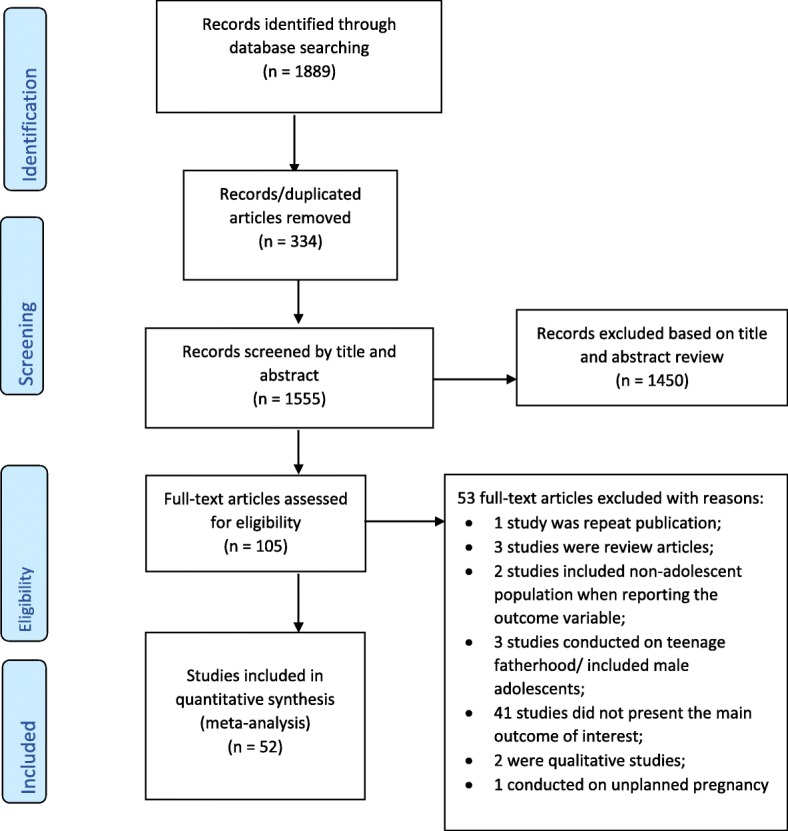
Flow diagram of the included studies for the systematic review and meta-analysis of prevalence and determinants of adolescent pregnancy in Africa

This systematic review and meta-analysis included published and unpublished studies conducted on adolescent pregnancy in Africa. A total of 1889 records were retrieved through electronic database searching. From these, 334 duplicated records were excluded, and from 1555 articles screened using their titles and abstracts, 1450 were excluded. One hundred five full-text articles were assessed for eligibility. From these, 53 full-text articles were excluded for *prior* criteria, and a total of 52 studies were included in the final quantitative synthesis (Fig. 1).

### Characteristics of included studies

Twenty-four African countries were represented in this review. From all, 18 (34.6%) of the studies were from West Africa [17, 19, 20, 22, 33, 35–37, 39, 48, 51–54, 58, 59, 67, 68], 19 (36.5%) were from East African countries [12, 13, 15, 16, 18, 21, 23, 24, 29, 30, 32, 38, 40, 42, 44–46, 69, 70], 7 (13.5%) from Central Africa [25, 47, 49, 50, 56, 57, 71], 6 (11.5%) from Southern Africa [31, 34, 43, 67, 72, 73] and 2 (3.8%) were from only one Northern African country (Egypt) [41, 55]. Almost all, 50 of the included studies were from Sub-Saharan African countries. The majority, 49 of the studies were published while only 3 studies were unpublished [16, 25, 50] (Table 1).

**Table 1:** Summary characteristics of studies included in the systematic review and meta-analysis of the prevalence and determinants of adolescent pregnancy in Africa

Authors & year	Study area, country	Sub-region	Study design and type	Study population	Range of ages of participants	Sample size	Response rate (%)	Outcome measured	Prevalence of adolescent pregnancy
Mathewos and Mekuria, 2018 [12]	Arba Minch town, Ethiopia	East Africa	Cross sectional, institution-based	School adolescent students	15 to 19	578	100	Ever pregnant	7.7
Akanbi F et al., 2016 [15]	Naguru Teenage Centre Kampala, Uganda	East Africa	Cross sectional, institution-based	Adolescent girls	13 to 19	384	82	Current pregnant	39.7
Sungwe, 2015 [16]	Zambia, 2007 DHS	East Africa	Cross sectional, community-based	Adolescent girls	15 to 19	1574	100	Ever pregnant	28.5
Kaphagawani and Kalipeni, 2017 [69]	Zomba district, Malawi	East Africa	Cross sectional, institution-based	Women visiting ANC	10 to 19	505	100	Current pregnant	37.1
Agbor et al., 2017 [47]	Health facilities in rural Cameroon	Central Africa	Retrospective study, institution-based	All deliveries during the study period	10 to 19	2343	77	Current pregnant	20.4
Malawi 2015–16 DHS, 2017 [45]	The 2015–16 DHS, Malawi	East Africa	Cross sectional, community-based	Adolescent girls	15 to 19	5263	100	Ever pregnant	29
Ethiopia 2016 DHS, 2017 [29]	The 2016 DHS, Ethiopia	East Africa	Cross sectional, community-based	Adolescent girls	15 to 19	3381	100	Ever pregnant	13
Jonas et al., 2016 [72]	South Africa	Southern Africa	Cross sectional, institution-based	School adolescent students	11 to 19	16,614	100	Ever pregnant	20.5
Garba et al., 2016 [48]	Aminu kano teaching hospital, Nigeria	West Africa	Retrospective study, institution-based	All deliveries during the study period	15 to 19	9312	100	Current pregnant	5.8
Zimbabwe 2015 DHS, 2016 [45]	The 2015 DHS, Zimbabwe	East Africa	Cross sectional, community-based	Adolescent girls	15 to 19	2199	100	Ever pregnant	21.6
Rwanda 2014–15 DHS, 2016 [44]	The 2014–15 DHS, Rwanda	East Africa	Cross sectional, community-based	Adolescent girls	15 to 19	2768	100	Ever pregnant	7.3
Lesotho 2014 DHS, 2016 [43]	The 2014 DHS, Lesotho	Southern Africa	Cross sectional, community-based	Adolescent girls	15 to 19	1440	100	Ever pregnant	19.1
Tanzania 2015–16 DHS, 2016 [42]	The 2015–16 DHS, Tanzania	East Africa	Cross sectional, community-based	Adolescent girls	15 to 19	2904	100	Ever pregnant	26.7
Beyene et al., 2015 [18]	Assosa general Hospital, Benishangul Gumuz region, Ethiopia	East Africa	Cross sectional, institution-based	Teenage females visiting hospital for health care services	13 to 19	783	98.3	Ever pregnant	20.4
Okigbo and Speizer, 2015 [70]	Five urban areas in Kenya, Kenya	East Africa	Cross sectional, community-based	Unmarried young women	15 to 19	750	100	Ever pregnant	8.1
Ngowa et al., 2015 [49]	Yaoundé general hospital, Cameroon	Central Africa	Retrospective study, institution-based	All delieveries during the study period	10 to 19	11,640	100	Current pregnant	2.84
Egbe et al., 2015 [71]	Buea health district, South West Region, Cameroon	Central Africa	Retrospective study, institution-based	Women visiting ANC	10 to 19	6564	100	Current pregnant	13.3
Schipulle, 2015 [50]	Three health centers in the central Gabon	Central Africa	Retrospective study, institution-based	All delieveries during the study period	12 to 19	1972	100	Current pregnant	23.7
Kenya 2014 DHS, 2015 [40]	The 2014 DHS, Kenya	East Africa	Cross sectional, community-based	Adolescent girls	15 to 19	5820	100	Ever pregnant	18.1
Egypt 2014 DHS, 2015 [41]	The 2014 DHS, Egypt	North Africa	Cross sectional, community-based	Adolescent girls	15 to 19	5185	100	Ever pregnant	10.9
Ghana 2014 DHS, 2015 [39]	The 2014 DHS, Ghana	West Africa	Cross sectional, community-based	Adolescent girls	15 to 19	1625	100	Ever pregnant	14.2
Zambia 2013–14 DHS, 2015 [38]	The 2013–14 DHS, Zambia	East Africa	Cross sectional, community-based	Adolescent girls	15 to 19	3625	100	Ever pregnant	28.5
Envuladu et al., 2014 [19]	Rural Community in Jos, Plateau State, Nigeria	West Africa	Cross sectional, community-based	Teenage girls	13 to 19	192	100	Ever pregnant	25.5
Brahmbhatt et al., 2014 [67]	Urban disadvantaged settings, South Africa	Southern Africa	Cross sectional, community-based	Adolescent girls	15 to 19	224	100	Ever pregnant	28.8
Brahmbhatt et al., 2014 [67]	Urban disadvantaged settings, Nigeria	West Africa	Cross sectional, community-based	Adolescent girls	15 to 19	229	100	Ever pregnant	24.1
Gambia 2013 DHS, 2014 [37]	The 2013 DHS, Gambia	West Africa	Cross sectional, community-based	Adolescent girls	15 to 19	2407	100	Ever pregnant	17.5
Sierra Leone 2013 DHS, 2014 [36]	The 2013 DHS, Sierra Leone	West Africa	Cross sectional, community-based	Adolescent girls	15 to 19	3878	100	Ever pregnant	27.9
Liberia 2013 DHS, 2014 [33]	The 2013 DHS, Liberia	West Africa	Cross sectional, community-based	Adolescent girls	15 to 19	2080	100	Ever pregnant	31.3
Namibia 2013 DHS, 2014 [34]	The 2013 DHS, Namibia	Southern Africa	Cross sectional, community-based	Adolescent girls	15 to 19	1906	100	Ever pregnant	18.6
Nigeria 2013 DHS, 2014 [35]	The 2013 DHS, Nigeria	West Africa	Cross sectional, community-based	Adolescent girls	15 to 19	7820	100	Ever pregnant	22.5
Fayemi et al., 2013 [68]	Ekiti state, Nigeria	West Africa	Cross sectional, community-based	Adolescent girls	13 to 18	400	100	Ever pregnant	25
Ugboma et al., 2012 [51]	Ebonyi state university teaching hospital, Nigeria	West Africa	Retrospective study, institution-based	All delieveries during the study period	13 to 19	8297	100	Current pregnant	5.5
Iklaki et al., 2012 [52]	University of Calabar Teaching Hospital, Calabar, Nigeria	West Africa	Retrospective study, institution-based	All delieveries during the study period	10 to 19	9906	100	Current pregnant	6.5
Ezegwui et al., 2012 [53]	Tertiary hospital in Enugu, Nigeria	West Africa	Retrospective study, institution-based	All delieveries during the study period	11 to 19	4422	100	Current pregnant	1.62
Amoran, 2012 [58]	Sagamu local government area, Ogun State, Nigeria	West Africa	Cross sectional, institution-based	All pregnant women attending the primary health care	10 to 19	225	100	Current pregnant	22.9
Nyarko, 2012 [22]	The 2008 DHS, Ghana	West Africa	Cross sectional, community-based	Adolescent girls	15 to 19	1037	100	Ever pregnant	10.2
Mchunu et al., 2012 [73]	South Africa	Southern Africa	Cross sectional, community-based	Youths	10 to 19	3123	96.4	Ever pregnant	19.3
Isa and Gani, 2012 [54]	Niger delta university teaching hospital, Bayelsa state, Nigeria	West Africa	Cross sectional, institution-based	All delieveries during the study period	13 to 19	1341	100	Current pregnant	6.2
Uganda 2011 DHS, 2012 [32]	The 2011 DHS, Uganda	East Africa	Cross sectional, community-based	Adolescent girls	15 to 19	2048	100	Ever pregnant	23.8
Rasheed et al., 2011 [55]	Sohag university hospital, Sohag, Egypt	North Africa	Retrospective study, institution-based	All delieveries during the study period	10 to 19	30,441	100	Current pregnant	7.5
Maduforo and Oluwatoyin, 2011 [59]	General hospital Ganye, Nigeria	West Africa	Cross sectional, institution-based	Women visiting ANC	10 to 19	106	100	Current pregnant	50.9
Alemayehu et al., 2010 [23]	The 2005 DHS, Ethiopia	East Africa	Cross sectional, community-based	Adolescent girls	15 to 19	3266	100	Ever pregnant	13.6
Tebeu et al., 2010 [56]	Referral maternity units in Cameroon	Central Africa	Retrospective study, institution-based	All delieveries during the study period	10 to 19	57,787	99.7	Current pregnant	14.23
Swaziland 2006–07 DHS, 2008 [31]	The 2006–07 DHS, Swaziland	Southern Africa	Cross sectional, community-based	Adolescent girls	15 to 19	1274	100	Ever pregnant	22.6
Iloki et al., 2004 [57]	Brazzaville university hospital, Congo	Central Africa	Cross sectional, institution-based	All delieveries during the study period	< 18	5204	100	Current pregnant	5.3
Fathi, 2003 [25]	The 1998 DHS, Cameroon	Central Africa	Cross sectional, community-based	Adolescent girls	15 to 19	1282	100	Ever pregnant	31.2
Eritrea 2002 DHS, 2003 [30]	The 2002 DHS, Eritrea	East Africa	Cross sectional, community-based	Adolescent girls	15 to 19	1129	100	Ever pregnant	23
Ayele et al., 2018 [13]	Deguwa Tembien district, Tigry, Ethiopia	East Africa	Case control, community-based	Adolescent girls	13 to 19	414	100	Current pregnancy	–
Izugbara, 2015 [17]	2008 DHS, Nigeria	West Africa	Cross sectional, community-based	Adolescent girls	13 to 19	6592	100	Current pregnancy	–
Philemon, 2007 [24]	Kinondoni Municipality, Dar-Es-Salaam, Tanzania	East Africa	Cross sectional	Adolescent girls	10 to 19	246	83	Current pregnancy	–
Kupoluyi et al., 2013 [20]	2003 DHS, Nigeria	West Africa	Cross sectional, community-based	Adolescent girls	10 to 19	7819	100	Ever pregnant	–
Gideon, 2013 [21]	2011 DHS, Uganda	East Africa	Cross sectional, community-based	Adolescent girls	15 to 19	2026	100	Ever pregnant	–

Forty one (78.8%) of the included studies were cross-sectional studies [12, 15–25, 29–46, 54, 57–59, 67–70, 72, 73], of which 18 studies were most recent DHS survey reports [29–46]. Ten (22.7%) were retrospective studies [47–53, 55, 56, 71] and one study [13] was case-control. Almost two thirds, 31 of the articles assessed the percentage of adolescent girls who begun childbearing, while 16 of the studies assessed the percentage of current pregnancy. Also, thirty two of the studies were community based [13, 16, 17, 19–23, 25, 29–46, 67, 68, 70, 73], while 19 were institution-based studies [12, 15, 18, 47–59, 69, 71, 72]. The sample size of the included studies ranged from a minimum of 106 in a study conducted in Nigeria (59) to maximum of 57,787 in a study conducted in Cameroon [56]. Overall, this review included a total of 254,350 study participants (Table 1). The study quality score of included studies ranged from 6 to 10, with the mean study quality score (+ standard deviation) of 8.48 + 1.57.

A meta-regression analysis was conducted since there was statistically significant heterogeneity, *I-square* test statistics less than 0.05. The purpose of the analysis was to identify the source of heterogeneity so that correct interpretation of the findings is made. However, the meta-regression analysis found no significant variable which can explain the heterogeneity. There was no statistically significant study level covariate: sample size, publication year, study quality score, sub-region, and publication status of included studies. Therefore, the heterogeneity can be explained by other factors not included in this review. (Table 2).

**Table 2:** Meta-regression analysis of the different study-level covariates to explain the sources of heterogeneity for meta-analysis of the prevalence and determinants of adolescent pregnancy in Africa

Variables	Coefficient	*P-value*
Publication year	0.24	0.653
Sample size	- < 0.001	0.672
East Africa	4.31	0.572
Southern Africa	12	0.266
West Africa	−3.88	0.560
Central Africa	−3.53	0.612
Unpublished studies	19.22	0.319

### Prevalence of adolescent pregnancy in Africa

**Fig. 2:**
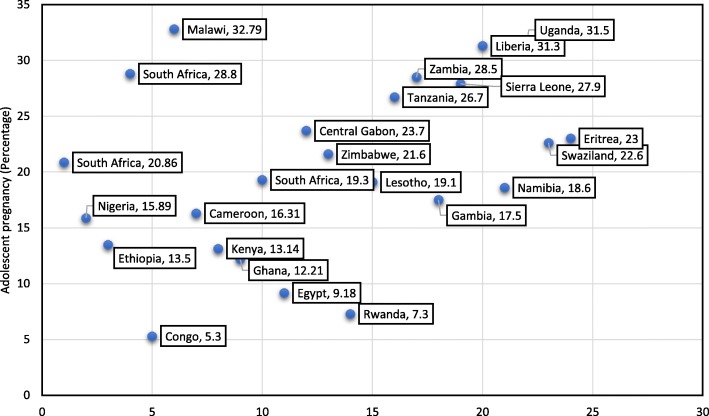
Distribution of pooled prevalence of adolescent pregnancy in 24 African countries, 2003 to 2018

**Fig. 3:**
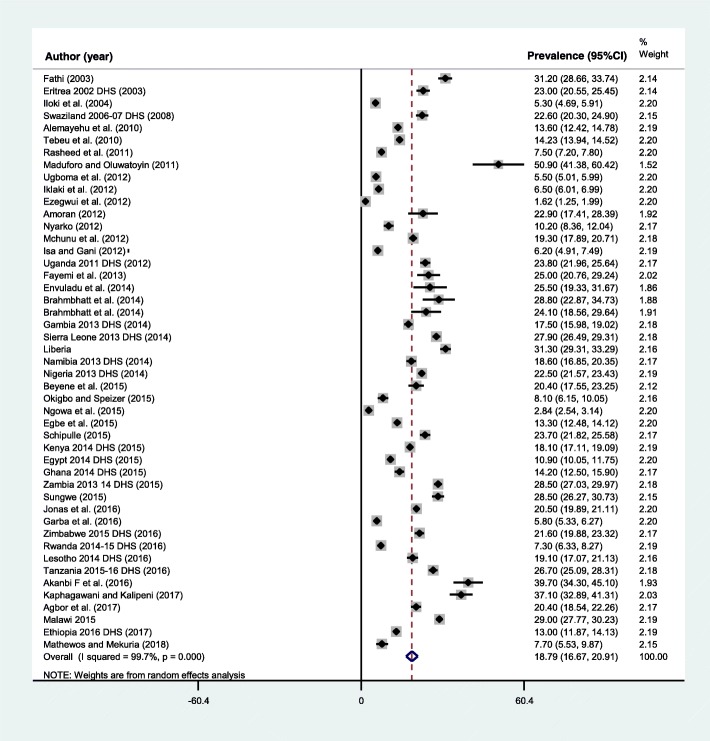
Prevalence of adolescent pregnancy in Africa, 2003 to 2018

The pooled prevalence adolescent pregnancy ranged from 1.62 to 51%, both in Nigeria [53, 59] (Fig. 2)**.** The prevalence was highest, 21.5% (95%CI: 17.3, 25.7) in the East African sub-region**,** followed by 20.4% (95%CI: 18.9, 21.7) in Southern Africa, 17.7% (95%CI: 14.1, 21.4) in West Africa, 15.8 (95%CI: 10.3, 21.3) in Central Africa, and the lowest was in Northern Africa, 9.2% (95%CI: 5.8, 12.5). Similarly, the pooled prevalence of adolescent pregnancy in Sub-Saharan African countries was 19.3% (95%CI: 16.9, 21.6). Overall, the pooled prevalence of adolescent pregnancy in Africa was 18.8% (95%CI: 16.7, 20.9) (Table 3). A significant heterogeneity of included studies in the meta-analysis was observed, *I*^*2*^ = 99.7%, *p* < 0.001. The Egger’s regression asymmetry test also showed significant publication bias, *p*-value < 0.001. After adjustment, the final pooled prevalence of adolescent pregnancy in Africa after the trim and fill analysis was 18.8% (95%CI: 16.7, 20.9) (Fig. 3).

**Table 3:** Subgroup analysis of the prevalence of adolescent pregnancy in Africa, 2003–2018

Subgroup	Number of studies	Total Sample	Prevalence (95%CI)	Heterogeneity	
				*I* ^*2*^	*p-value*
By Sub-region					
East Africa	16	36,977	21.5 (17.3, 25.7)	99.1	< 0.001
Southern Africa	6	24,581	20.4(18.9, 21.7)	73.7	0.002
West Africa	16	53,277	17.7 (14.1, 21.4)	99.6	< 0.001
Central Africa	7	86,792	15.8 (10.3, 21.3)	99.8	< 0.001
Northern Africa	2	35,626	09.2 (05.8, 12.5)	98.2	< 0.001
Sub Saharan African countries	45	201,627	19.3 (16.9, 21.6)	99.7	< 0.001
By study design					
Cross-sectional (including survey studies)	37	94,569	21.3 (18.6, 24)	99.2	< 0.001
Retrospective study	10	142,684	10.1 (06.9, 13.2)	99.8	< 0.001
By study type					
Community-based	28	68,829	20.9 (18.2, 23.7)	98.9	< 0.001
Institution-based	18	168,424	15.2 (12.5, 17.9)	99.7	< 0.001
By type of the document					
DHS final report	18	56,752	20.9 (17.4, 24.3)	99.1	< 0.001
Research article	29	180,501	17.2 (14.9, 19.5)	99.7	< 0.001
By publication status					
Unpublished studies	3	4828	27.7 (23.3, 32.2)	91.7	< 0.001
Published studies	44	232,425	18.2 (16, 20.3)	99.7	< 0.001
By publication year					
Before 2015 (during MDG period)	35	189,562	18.2 (15.9, 20.5)	99.7	< 0.001
2016 to 2018 (post MDG)	12	47,691	20.5 (15.1, 25.9)	99.6	< 0.001
By quality score					
Low score	22	164,964	16.3 (13.6, 18.9)	99.7	< 0.001
High score	25	72,289	20.8 (17.3, 24.4)	99.4	< 0.001
Total	47	237,253	18.8 (16.7, 20.9)	99.7	< 0.001

A higher (21.3%) prevalence of adolescent pregnancy was observed among studies conducted using the cross-sectional design compared to 10.1% in retrospective studies. Similarly, the prevalence using community-based studies was 20.9% (95%CI: 18.2, 23.7) and it was 15.2% (95%CI: 12.5, 17.9) using the institution-based studies. The result of the eighteen DHS reports showed a pooled prevalence of adolescent pregnancy of 20.9% (95%CI: 17.4, 24.3). Also, using the high-quality score studies, the prevalence of adolescent pregnancy in Africa was 20.8% (95%CI: 17.3, 24.4) and it was 16.3% (95%CI: 13.6, 18.9) for low quality score studies. The prevalence of adolescent pregnancy prior to the end of the Millennium Development Goals (MDGs) period (2003 to 2015 in the current review) was 18.2% (95%CI: 15.9, 20.5), which rose to 20.5% (95%CI: 15.1, 25.9) during the post MDG (2016 to 2018) (Table 3**).**

### Factors associated with adolescent pregnancy

#### Sociodemographic characteristics

**Fig. 4:**
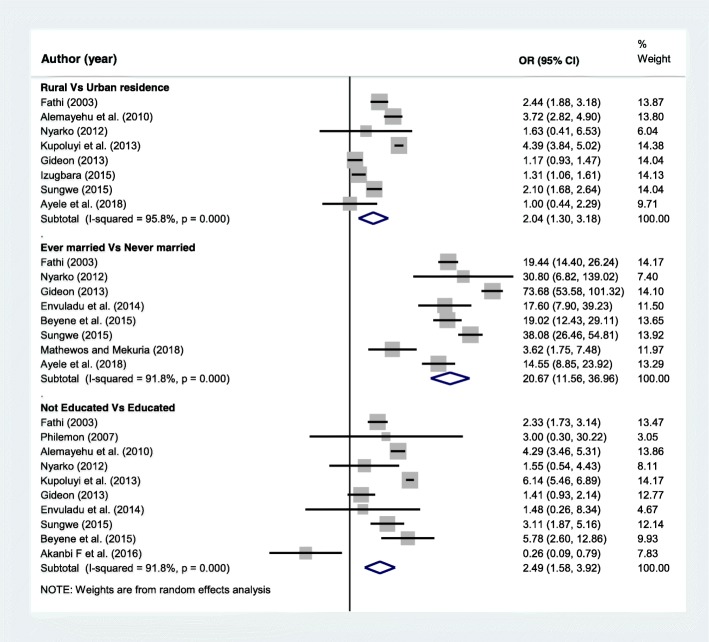
Forest plot of odds ratio for the association of selected sociodemographic characteristics and adolescent pregnancy in Africa

The sociodemographic factors included in this analysis were the place of residence, marital status and educational status of adolescent girls. A separate analysis was conducted for each variable. A total of 8 articles [13, 16, 17, 20–23, 25] were included to determine the association of place of residence and adolescent pregnancy. Five of the included studies [16, 17, 20, 23, 25] found significant association while the rest three articles [13, 21, 22] showed non-significant association between residence and adolescent pregnancy. The final pooled meta-analysis showed that adolescents who reside in rural areas were two times more likely to be pregnant than adolescent girls who live in urban areas, OR = 2.04 (95%CI = 1.3, 3.18). Additionally, A total of 8 articles [12, 13, 16, 18, 19, 21, 22, 25] were included to assess the association of marital status and adolescent pregnancy. The pooled meta-analysis showed that ever married adolescents were more than twenty times more likely to start childbearing during adolescence age than adolescents who were never married, OR = 20.67(95%CI = 11.56, 36.96) (Fig. 4).

Similarly, ten articles [15, 16, 18–25] were also included to determine the association of educational status of adolescent girls and experience of pregnancy. From this, five articles [16, 18, 20, 23, 25] found higher pregnancy rate among adolescents who had no education, while one article found the opposite [15]. The rest four researches [19, 21, 22, 24] showed non-significant association. However, the final pooled meta-analysis using data from the ten articles found that adolescent girls who are not attending school are more than two times more likely to start childbearing than those who are in school, OR = 2.49 (95%CI = 1.58, 3.92) (Fig. 4).

**Fig. 5:**
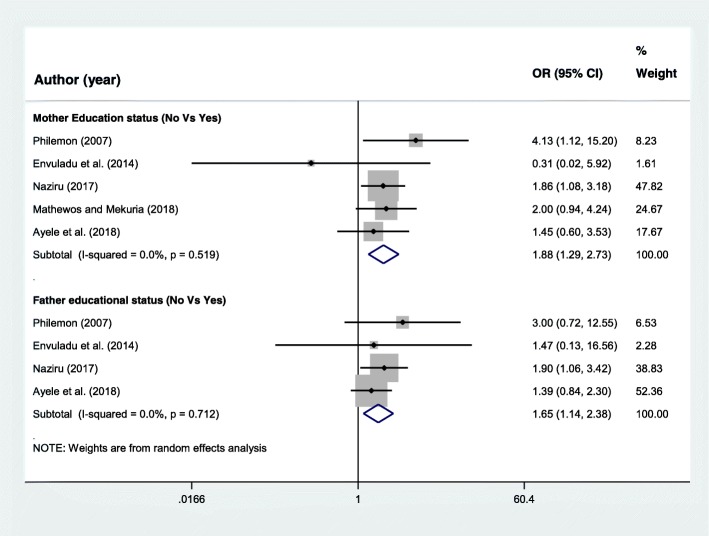
Forest plot of odds ratio for the association of family educational characteristics and adolescent pregnancy in Africa

Family educational characteristics A total of five research articles [12–14, 19, 24] were included to assess the association of mother’s educational status and adolescent pregnancy. From this, two studies [14, 24] found significant association and the rest three articles [12, 13, 19] found no association. However, the final pooled meta-analysis showed that adolescents with mother’s educational status of not educated were almost two times more likely to start childbearing during adolescence period than their counterparts, OR = 1.88 (95%CI = 1.29, 2.73) (Fig. 5).

Four articles [13, 14, 19, 24] were also included to determine the association of father’s educational status and adolescent pregnancy. Even though all included studies independently had non-significant association, the pooled meta-analysis showed statistically significant association. Adolescents with father’s educational status of not educated were 1.65 times more likely to start childbearing than those whose father were educated, OR = 1.65 (95%CI = 1.14, 2.38) (Fig. 5).

### Parent to adolescent communication on SRH issues

**Fig. 6:**
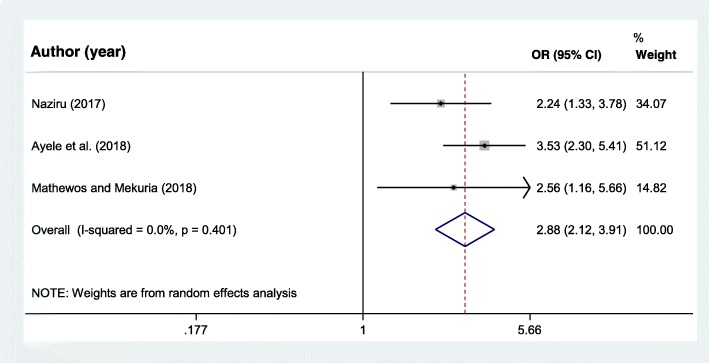
Forest plot of the odds ratio for the association of lack of sexual and reproductive health communication between adolescents and parents and adolescent pregnancy in Africa

Three research articles [12–14] were included to assess the association between parent to adolescent communication on SRH and adolescent pregnancy. The final pooled meta-analysis showed that adolescents who had no open discussion or communication on SRH issues with their parents were almost three times more likely to start childbearing, OR = 2.88 (95% = 2.12, 3.91) (Fig. 6).

## Discussion

This systematic review and meta-analysis was conducted to estimate the prevalence and determinants of adolescent pregnancy in Africa using the available published and unpublished studies. Adolescent pregnancy is considered a risk factor for adverse maternal and neonatal outcomes [2]. A WHO report showed that pregnancy and childbirth complications are the second commonest causes of death among adolescent girls [5].

This study found a higher prevalence of adolescent pregnancy in Africa compared to other low- and middle-income countries (LMIC). The pooled prevalence of adolescent pregnancy in Sub-Saharan African countries was 19.3%, higher than the overall prevalence of adolescent pregnancy in Africa (18.8%). This finding is much higher compared to 6.4% (× 3) in Latin America, 4.5% (× 4) in Southeastern Asia and 0.7% (× 26) in Eastern Asia [74]. The inaccessibility of contraceptive services, the unfavorable attitude of the community towards the adolescent contraceptive use, poor knowledge of adolescents of the SRH) issues and widespread sexual violence in developing countries are some of the reasons for the higher prevalence of adolescent pregnancy in Africa [75]. In addition, the prevalence of unmet need for contraceptives among adolescents in Sub-Saharan African countries is high, resulting in a high rate of unwanted pregnancy in the region [76]. Also, half of the adolescent pregnancy occurring among 15 to 19 years old girls in developing countries are unintended [77]. Improvement of the knowledge of adolescents towards the SRH issues, increasing the contraceptive access and use among young people, and reducing child marriage is important to prevent adolescent pregnancy and reduce its poor maternal and neonatal outcomes [75].

Variations in the rate of adolescent pregnancy were observed in different sub-regions of Africa, the highest in East Africa (21.5%) and lowest in Northern Africa (9.2%). Sociocultural, environmental and economic factors, resulting in differences in the access to the already inadequate adolescent sexual and reproductive health services can be mentioned as possible reasons for the observed disparities. These factors are also mentioned as reasons for a high level of adolescent pregnancy in Sub Saharan Africa [26, 78]**.** Evidence also showed that adolescent pregnancy is more likely to occur in poor countries and communities with poor education and employment opportunities [79].

Despite several progresses made by the governmental and non-governmental organizations, the global rate of adolescent pregnancy and birth rate is still high [80]. This study also showed an increasing level of adolescent pregnancy in Africa in the years 2016 to 2018 (20.5%) than studies conducted before 2015 (18.2%). This could be related to a higher detection rate in the recent years because diagnosis of pregnancy is earlier and surer and more rural areas are accessing these tests more than before. Researchers are penetrating the rural areas more, in more coordinated and multidisciplinary fashion [13, 16, 17, 20–23, 25]. Moreover, the United Nations Population Fund (UNFPA) report on adolescent pregnancy also showed that the percentage of adolescent pregnancies will increase globally by 2030, particularly in the Sub Saharan African countries [80]. The increasing number of the adolescent population in the continent can be mentioned as a reason for the high rate of adolescent pregnancy [80]. This calls for efforts to address the sexual and reproductive health needs of adolescent girls to achieve the SDG targets on reduction of maternal mortality.

The increasing prevalence of adolescent pregnancy in Africa is one of the reasons for the high rate of maternal and child morbidity and mortality on the continent. Moreover, 99 % of maternal deaths of women aged 15 to 19 years occur in LMIC, particularly in Sub Saharan African countries [81]. Adolescent pregnancy is also a major contributor to an intergenerational cycle of poverty and poor health outcomes. Therefore, emphasis should be given to the prevention of adolescent pregnancy through improvement of contraceptive access, adolescent-friendly health services, and sexuality education [75]. Studies found that educational programs aimed at reducing sexual risk behaviors and prevention of pregnancy among young people can effectively reduce the pregnancy rates among teenagers [82]. Also, programs aimed at abstinence-centered sexuality education are also effective in preventing adolescent pregnancy [83].

This review also assessed the association of selected variables with adolescent pregnancy. Adolescents who live in rural areas were more likely to start childbearing than adolescents in urban areas. This could be because of the lack of educational opportunities, poverty and limited access to SRH services in ssrural than urban areas [84, 85]. A systematic review of studies to assess factors associated with adolescent pregnancy in LMIC also found similar findings [78]. This calls the design of health services specifically designed for rural adolescents. Future researchers should also address the gap of studies on the needs of adolescent girls and possible interventions needed to reduce adolescent pregnancy in rural areas.

The current study also found that adolescents who were ever married were more likely to start childbearing than those who were never married. Even though several national and international laws forbid early marriage, the practice is still common in many countries, particularly in African countries [86]. A recent world bank report showed investment in reducing child marriage can result in substantial reduction in population growth, and improves child health and even reduces the economic cost [87, 88]. Furthermore, early marriage is associated with several SRH complications. For example, problems like sexually transmitted diseases, complications during childbirth, including obstetric fistula are common among adolescent who are married before their eighteenth birthday [89]. Early marriage also exposes to high fertility and lower school attainment among adolescent girls [84]. Despite this, Sub-Saharan Africa is known for the highest proportion of adolescent girls who are married. For instance, more than one fourth (28%) of female adolescents aged 15 to 19 in West and Central Africa and 26.6% in Sub-Saharan Africa were currently married in 2010 [84].

The findings of this study and available evidence suggest that investment in ending child marriage is important not only to reduce adolescent pregnancy and related complications, but also to improve the economic development of a country. Therefore, adherence to the available legal frameworks against child marriage will help countries to achieve the national and international targets. Moreover, law enforcement to protect the sexual and reproductive health and human rights of adolescent girls is essential to end child marriage and adolescent pregnancy [84].

This study also found that adolescents who are not attending school are more likely to get pregnant or start childbearing than those who are in school. This may be related to the empowerment of adolescents attending school with the necessary skills to prevent pregnancy. Adolescents who are out of school are denied access to comprehensive sexuality education and skills needed to negotiate sexuality and reproductive options and prevent pregnancy. This could also justify the high rate of adolescent pregnancy in sub Saharan African countries. Because, the UNFPA report showed that almost one third of adolescents in Sub-Saharan Africa are out of school [84]. Furthermore, educated women are better informed of their basic SRH rights and are able to make better decisions to protect their health. Similar findings were found in LMIC [78]. Similarly, this review found that adolescent with educated parents, either father or mother, were less likely to start childbearing during young age than those with no parent education.

Age appropriate sexual health education is important for adolescent to develop safe sexual and reproductive health and to prevent adolescent pregnancy [90]. This review also found that adolescents who had discussions with their parents are less likely to start childbearing than those who had no discussion. Open discussion about sexuality among children and parents in homes is important to prevent adolescent pregnancy [91]. Moreover, previous studies have shown that risk reduction education programs and parental support are effective in reducing adolescent pregnancy [92, 93], lower risk behaviors, and improve healthy sexual decision making including consistent and correct use of condoms [94]. This finding suggests the importance of design of programs which facilitate parent to child communication on sexuality issues, especially in resource limited areas where access to SRH information is very limited. But, there still exists a controversy about the extent of effect of parents on sexual health decisions of adolescents [95].

The factors affecting adolescent pregnancy are not limited to sociodemographic characteristics. Factors like employment attainment [23], lower economic status [13], living arrangement, the sex of the household head [17], history of maternal teenage pregnancy [13], knowledge toward SRH issues, family planning use [18], presence or absence of sexuality education in schools, and substance use [12] also affects adolescent pregnancy.

This review has certain strengths and limitations. It included a large number of published and unpublished studies conducted in Africa. The PRISMA guideline was strictly followed in all steps of the systematic review and meta-analysis. Also, the most recent DHS reports of African countries were retrieved from the official DHS program website [28]. The inclusion of population-based studies (DHS surveys) improves the generalizability of the findings since they used validated tools to measure the outcome variable. But, prevalence data collected in the clinic-based studies may have introduced bias since the population in these studies may not represent the general population. On the other hand, the fact that self-performed abortions are becoming common, especially when performed early, make it difficult to determine the true prevalence of adolescent pregnancy. Abortifacients like misoprostol tablets are poorly restricted in many African countries [96]. Additionally, only studies published in the English language were included. Also, due to the absence of articles from some of them, this study didn’t include all African countries. The quality assessment also showed evidence of poor quality, and this may affect the findings. However, we conducted proper subgroup analysis by the quality of included studies. Additionally, most of the articles included in this review assessed the sociodemographic characteristics as the main factor and there were limited studies which presented the association of other variables like social, economic andSRH issues with adolescent pregnancy. For this reason, this review mainly included those studies which presented the selected sociodemographic factors discussed above. Future review studies which elucidate the association of adolescent pregnancy with other factors like social and economic factors, substance use, peer pressure, knowledge regarding SRH issues including family planning are important. Also, this review didn’t include qualitative studies on the reasons for adolescent pregnancy. Other behavioral, environmental and health care system variables that affect adolescent pregnancy were also not addressed, and this ispossible future research area.

## Conclusions

Almost one-fifth of adolescent girls in Africa gets pregnant. Wide differences in rates were observed across the different sub-regions of Africa, the highest being in the Eastern African region. Countries should work towards preventing adolescent pregnancy through school and community-based family life education that promotes abstinence and safe sexual practice. Better access to contraceptive information and the use of contraceptive methods by adolescent girls to avoid unwanted pregnancy should be encouraged. Special focus should be given to the diverse sexual and reproductive health needs of adolescents by policymakers, population planners, researchers and healthcare workers.

This review also found different sociodemogaphic factors associated with adolescent pregnancy. Adolescents from rural residence, ever married, not educated, no mother’s education, no father’s education, and lack of parent to child communication on SRH issues were more likely to start childbearing. Future intervention programs for prevention of adolescent pregnancy need to target the identified factors. Moreover, further large-scale review studies are also needed to investigate environmental, behavioral and other social, economic and family related factors associated with adolescent pregnancy and thereby to plan and effect interventions. Experimental studies aimed at reducing unwanted pregnancy among adolescent girls in resource-limited settings are also recommended.

## Abbreviations

CI: Confidence Interval
DHS: Demographic and Health Survey
LMIC: Low- and Middle-Income Countries
MDG: Millennium Development Goal
OR: Odds Ratio
SDG: Sustainable Development Goal
SRH: Sexual and Reproductive Health
WHO: World Health Organization
